# Tumeur noire de l’ombilic

**DOI:** 10.11604/pamj.2019.33.319.15697

**Published:** 2019-08-22

**Authors:** Fatima-Zahra Agharbi

**Affiliations:** 1 Centre Hospitalier Régional Tétouan, Tétouan, Maroc

**Keywords:** Nævus, ombilic, congenital, Naevus, umbilicus, congenital

## Abstract

Pigmented nevi, still called melanocytic nevi or more improperly “naevus naevocellulaires” are benign melanocytic tumors characterized by a proliferation of melanocytes near the dermoepidermal junction. They are grouped in clusters or in theca cells that differentiate them from normal melanocytes. They occur at birth in approximately 1% of newborns with no sex predominance. Their occurrence is mostly sporadic, although rare family forms have also been reported. They can be separated in an arbitrary manner according to their diameter on the day of the birth: small congenital nevi <1.5cm, medium-sized 1.5 to 20cm and large-sized >20cm. Umbilical nevi are rare. We here report an interesting case of atypical and rare congenital nevus. The study involved a young girl presenting with pigmented umbilical nodule occurred at birth which had recently become problematic. We suspected a nevus, a melanoma or an endometriosis on the basis of the clinical appearance of this nodule. Resection was performed. The diagnosis of benign melanocytic tumor was confirmed.

## Image en médecine

Les nævus pigmentaires, encore appelés nævus mélanocytaires ou plus improprement « nævus nævocellulaires » sont des tumeurs mélanocytaires bénignes caractérisées par une prolifération de mélanocytes à proximité de la jonction dermoépidermique, avec un regroupement en amas ou thèques qui les différencient des mélanocytes normaux. Les nævus congénitaux sont présents dès la naissance et surviennent chez environ 1% des nouveau-nés sans prédominance de sexe. Leur survenue est très majoritairement sporadique, bien qu'existent aussi de rares formes familiales. Ils peuvent être séparés de manière arbitraire en fonction de leur plus grand diamètre le jour de la naissance: petits nævus congénitaux si <1,5cm, moyens de 1,5 à 20cm et grands si > à 20cm. L'ombilic est une localisation rare de naevus. Nous rapportons un cas de nævus congénital dont la localisation atypique et rare nous a paru intéressante à rapporter. Il s'agit d'une jeune fillette qui consultait pour un nodule pigmenté de l'ombilic remontant à la naissance devenu gênant récemment. Devant cet aspect clinique nous avons évoqué: un nævus, un mélanome ou une endométriose. Une exérèse a été réalisée confirmant le diagnostic de nævus.

**Figure 1:**
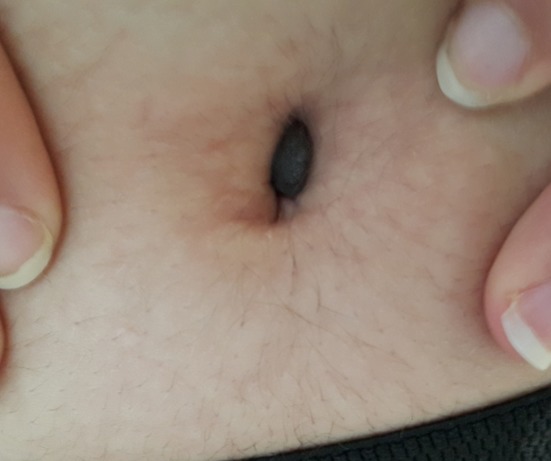
Nodule pigmenté de l'ombilic

